# Sustainable removal of methylene blue from wastewater using silica sand coated with Al_2_O_3_ nanoparticles: a comparative study of batch and fixed bed column reactors

**DOI:** 10.1039/d5ra07804k

**Published:** 2025-12-17

**Authors:** Erfan Burhan Hussein, Farouk Abdullah Rasheed

**Affiliations:** a Department of Water Resources Engineering, College of Engineering, University of Sulaimani Sulaymaniyah 46001 Kurdistan Region Iraq erfan.hussein@univsul.edu.iq farouk.rasheed@univsul.edu.iq

## Abstract

This research addresses the challenge of effectively removing methylene blue (MTB) dye from wastewater, given its high solubility and stubborn resistance to biodegradation. Iraqi silica sand coated with synthesized aluminum oxide nanoparticles (SSC–Al_2_O_3_) was investigated as a novel adsorbent for removing MTB molecules. This study demonstrated the adsorption capacity of SSC–Al_2_O_3_ in a batch configuration and predicted the breakthrough curves in a fixed-bed column reactor. At the ideal pH (6), under an agitation speed of 200 rpm and 75 minutes, isothermal equilibrium adsorption achieved 95.33% adsorption efficiency. Adsorption isotherms followed the Freundlich isotherm type. Experiments were carried out applying fixed bed columns with bed heights of 4 cm (78.5 g), 7 cm (137.4 g), and 10 cm (196.3 g) with a flow rate of 10 L h^−1^. The bed height of (10 cm) offered the longest breakthrough time and the least steep breakthrough curve. Higher flow rates decreased the adsorption capacity and yielded steeper breakthrough curves. FTIR analysis of SSC–Al_2_O_3_ showed that the hydroxyl, carboxyl, and amine functional groups were involved in bonding between MTB dye and the sorbent. This shows that SSC–Al_2_O_3_ has excellent ability in batch and continuous flow systems to treat industrial wastewater containing MTB dye.

## Abstract 2

This research addresses the challenge of effectively removing methylene blue (MTB) dye from wastewater, given its high solubility and stubborn resistance to biodegradation.

## Graphical abstract

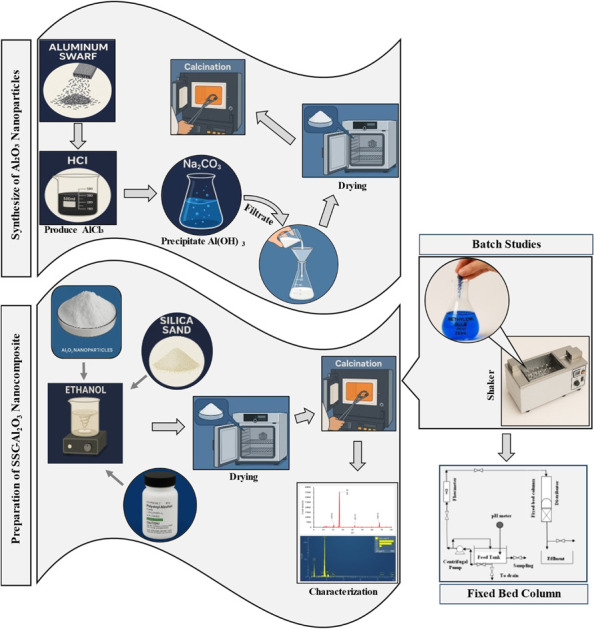

## 1 Introduction

Rapid industrialization and continued population growth have increasingly strained global freshwater resources and contributed to decline water quality.^1^ Industrial effluents are a major source of environmental pollution, with around one-third of industrial wastewater discharged without sufficient treatment.^2^ Among these pollutants, synthetic dyes represent one of the most persistent and hazardous groups due to their extensive use in pharmaceuticals, textiles, cosmetics, leather tanning, paper printing, and food processing industries.^3^ Even at low concentrations, dyes reduce water clarity, suppress oxygen solubility, hinder sunlight penetration, and disrupt photosynthetic activity in aquatic environments.^4^ Their stable aromatic molecular structures confer high chemical resistance and non-biodegradability, enabling prolonged environmental persistence and bioaccumulation.^5,6^ The continuous release of dye-laden wastewater into natural water bodies has been shown to cause sever ecological damage and negatively affect human health, with approximately 3.1% of global annual mortality linked to unsafe water conditions.^7–10^

Methylene blue (MTB) is a widely used basic cationic dye applied in textiles, leather, paper production, microbiological staining, aquaculture, and various pharmaceutical applications.^11^ Typical industrial effluents contain MTB concentrations ranging from 10 to 200 mg L^−1^, sufficient to generate intensely colored wastewater due to its exceptionally high molar absorption coefficient (∼8.4 × 10^4^ L mol^−1^ cm^−1^ at 664 nm).^12,13^ Once discharged, MTB significantly reduces sunlight penetration, lowers dissolved oxygen levels, and inhibits the photosynthesis of aquatic flora, ultimately threatening aquatic ecosystem stability.^12,14^ Studies have reported pronounced toxicity to aquatic organisms, including *Daphnia magna* (no observed effect concentration (NOEC) = 4.7 µg L^−1^), fish, and beneficial microbes, where MTB exposure damages gills, tissues, and vital organs.^12,14,15^ Due to its non-biodegradability and high environmental persistence, MTB can bioaccumulate in aquatic food chains, contributing to human exposure through contaminated water and fish consumption.^16–18^

At higher doses (>7 mg kg^−1^), MTB poses significant health risks to humans, including respiratory complications, gastrointestinal disorders, cardiovascular effects, methemoglobinemia, neurological damage, and tissue necrosis.^19–23^ Chronic exposure has been associated with increased cancer risk and sever systemic toxicity.^14,22^ Although MTB is considered safe at therapeutic doses (<2 mg kg^−1^) and is used in clinical applications such as photodynamic therapy, antimicrobial treatment, and the management of methemoglobinemia, it can act as a potent monoamine oxidase inhibitor, potentially inducing serotonin syndrome and teratogenic effects when improperly administered.^14,15^ Given that textile and related industries account for approximately 67% of global dyestuff consumption and discharge nearly 120 m^3^ of wastewater per ton of fiber produced,^14^ uncontrolled release of MTB-contaminated effluents remains a critical environmental challenge. Therefore, preventing MTB discharge and developing efficient treatment technologies are essential for mitigating ecological harm and protecting human health.^24^

Different approaches have been used to eliminate MTB from the effluents: coagulation, chemical oxidation, membrane filtration, and biological treatment.^25^ Nevertheless, these methods are not very efficient in removing traces of dyes because of their complexity, high cost, and inability to remove trace amounts of dyes. In contrast, adsorption is one of the most promising techniques for dye removal because it is simple, low cost, and has high efficiency.^26,27^ Furthermore, adsorption processes are suitable for a wide range of operational conditions, which could experience adjustments to improve performance through adsorbent regeneration. Moreover, adsorption has a high removal potential for dyes, even at low concentrations,^28^ and this makes it an attractive process for treating dye-containing industrial wastewater. Materials like activated carbon and nanoadsorbents have received considerable attention because of their active adsorption sites and suitable surface areas. However, the costly operation of these materials limits their broad use in industries.^29^ To overcome this challenge, researchers are investigating novel compounds with enhanced surface properties and selectivity upon adsorption in attempts to improve their binding abilities and broad applicability. In particular, increasing attention is being dedicated to the natural resource materials with these advantages, leading further to their enhancement as efficient and cost-effective adsorbents.^30^ However, coating rich silica sand, which is an abundant low-cost natural resource in Iraq, with synthesized Al_2_O_3_ nanoparticles synthesized from the industrial aluminum swarf produces a highly effective nanocomposite adsorbents and approach to capture dyes from industrial wastewater due to its scalability and availability. In addition to this, industrial aluminum swarf management could also serve as a precursor for the synthesis of these nanoparticles, offering a novel and green approach to the production of adsorbent materials.

Various adsorption techniques can be utilized to examine the mass transfer between an adsorbate (pollutant) and an adsorbent, including batch studies, continuous moving bed mechanisms, continuous fixed bed mechanisms, continuous fluidized bed systems, and pulsed bed systems. Batch adsorption is commonly employed in small-scale studies, however its applicability in large-scale industrial wastewater treatment is limited. The fixed-bed column system is considered more practical and scalable, providing continuous operation and enhanced pollutant removal efficiency. The analysis of fixed-bed adsorption performance can be conducted using breakthrough curves, which represent the concentration of pollutants in the effluent over time.^31^ Fixed-bed operation is affected by numerous variables, such as equilibrium adsorption (isotherm and capacity), kinetic (diffusion and convection coefficients), hydraulic hold-up, column geometry, and flow distribution within the column.^32^ Fixed-bed column adsorption presents several advantages over batch adsorption: (i) it simplifies operation and monitoring, aiding in transition from laboratory to industrial applications; (ii) it allows for more rapid treatment of contaminated water; and (iii) it improves effluent quality while maintaining high adsorption capacity. Conventional fixed-bed packing adsorbents, including natural clay, zeolite, and activated carbon, exhibit specific limitations despite their advantages. Clay and zeolite are classified as non-renewable mineral resources. In contrast, activated carbon is characterized by low selectivity, high production costs, and difficulties associated with regeneration and reuse. The advancement of sustainable, economical, and efficient fixed-bed packing adsorbent is a significant research focus.^33^

Recent studies have explored various methods for recovering alumina from aluminum waste. However, many of these methods produce micro-scale alumina with limited surface area. This work contributes to the advancement of sustainable wastewater treatment technologies by building on our previous research, which demonstrated the successful synthesis of Al_2_O_3_ nanoparticles from aluminum swarf waste and their effective use in MTB dye adsorption, along with post-adsorption applications for soil stabilization.^34^ The present study introduces three key innovations that distinguish it from previous studies. First, the Al_2_O_3_ nanoparticles used in this research were sustainably synthesized from industrial aluminum swarf waste, providing a circular-economy approach that reduces production cost and environmental burden compared with conventional chemical synthesis routes. Second, these nanoparticles were used to coat a naturally rich and abundantly available silica sand from Iraq, producing a low-cost, scalable nanocomposite (SSC–Al_2_O_3_) that has not been previously reported in the literature. This material uniquely combines the high affinity of Al_2_O_3_ nanoparticles with the mechanical stability, availability, and hydraulic suitability of natural silica sand for column applications. Third, the study integrates both batch and pilot-scale fixed-bed column performance to evaluate the practical applicability of this sustainable adsorbent under conditions that simulate industrial wastewater treatment. Unlike previous works that rely only on small-scale tests, the present study generates fixed-bed breakthrough data, models adsorption behavior, and assesses the material's feasibility for continuous-flow systems. Together, these innovations demonstrate a sustainable synthesis route, a regionally available support material, and a pilot-scale adsorption evaluation, establishing the SSC–Al_2_O_3_ nanocomposite as a practical and scalable option for MTB dye removal.

## 2 Materials

**Fig. 1:**
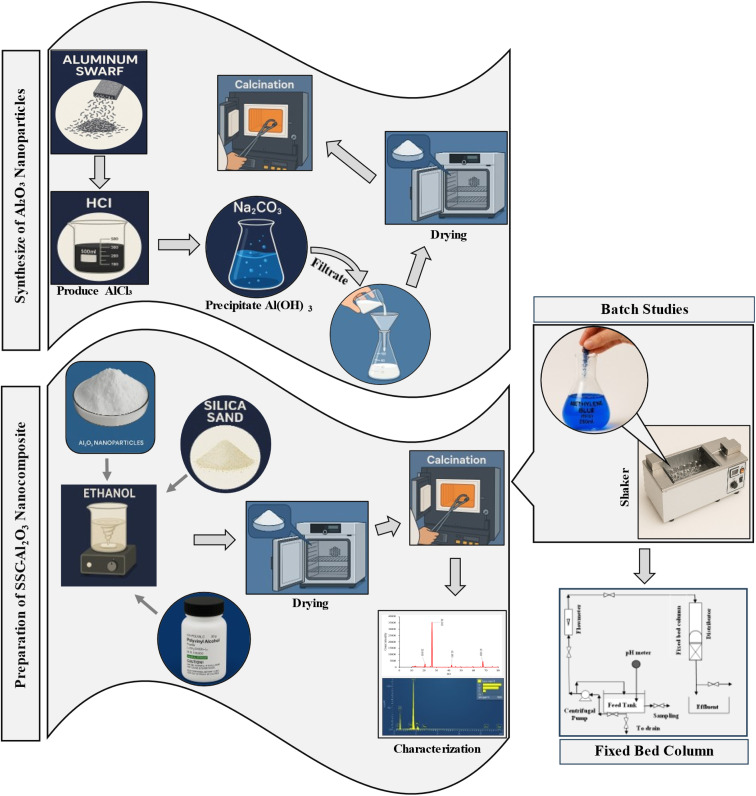
Graphical representation of the study methodology.

This study investigates the enhanced removal of MTB dye from wastewater using a nanocomposite adsorbent developed from industrial byproducts. Al_2_O_3_ nanoparticles were used to coat naturally abundant silica sand, resulting in the formation of SSC–Al_2_O_3_ nanocomposite. They produced nanocomposite was characterized using various analytical techniques to evaluate its surface morphology, structural, and chemical properties. The adsorption performance of the nanocomposite was initially evaluated using batch studies to optimize operational parameters. Subsequently, a pilot-scale fixed-bed column reactor was designed and implemented to evaluate the nanocomposite's adsorption capacity under continuous flow conditions, aiming to assess its feasibility for industrial scale wastewater treatment applications. Fig. 1 provides a schematic representation of the overall experimental procedure employed in this study.

### 2.1 MTB dye solution

MTB dye powder (C_16_H_18_N_3_SCl), taken from the inorganic laboratory, was used to prepare 1000 mg L^−1^ of stock solution. 1 g of MTB dye powder mass was weighed and agitated with 100 mL of deionized water. Once a uniform solution was reached, dilution was applied by the adding deionized water up to 1000 mL. The desired normality of 50 mg L^−1^ was obtained after diluting a limited volume of concentrated solution with deionized water.^35^ At the end of the adsorption process, the equilibrium concentration of MTB in the aqueous solution was measured using a UV spectrometer. The specific wavelength at which MTB absorbs UV light most significantly is 664 nm.

### 2.2 Preparation of SSC–Al_2_O_3_

Rich silica sand (RSS) was obtained from the Rutba location in Anbar city, with coordinates of 33°34′49.9″N 40°03′28.5″E, and was washed with deionized water to remove impurities, then dried in an oven at 70 °C for 2 h. Particles of RSS were sieved to obtain a seize diameter of 0.6 to 0.9 mm. Aluminum swarf was dissolved in concentrated hydrochloric acid (HCl) to generate an aluminum chloride (AlCl_3_) solution (see eqn (1)), which then served as the Al_2_O_3_ nanoparticle synthesis precursor. After the complete dissolution of the swarf, a gradual addition of sodium carbonate (Na_2_CO_3_) was performed to the solution; the addition resulted in the precipitation of aluminum hydroxide (Al(OH)_3_) (see eqn (2)). The obtained precipitate was filtered through the Whatman 42 filter paper and washed thoroughly with deionized water to remove excess salts or impurities. The precipitate was filtered off, dried, and calcined at 600 °C, yielding Al_2_O_3_ nanoparticles from Al(OH)_3_ (ref. 34 and 36) (see eqn (3)).

1 2Al (s) + 6HCl (aq) → 2AlCl_3_ (aq) + 3H_2_ (g)

2 2AlCl_3_ (aq) + 3Na_2_CO_3_ (aq) + 3H_2_O (l) → 2Al(OH)_3_ (s) + 6NaCl (aq) + 3CO_2_ (g)

3
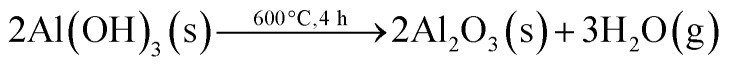


The mass of 0.6 g of Al_2_O_3_ nanoparticles is dispersed in 75 mL of ethanol using a magnetic stirrer or a sonicator for uniform dispersion. The Al_2_O_3_ nanoparticles suspension keeps stirring continuously when the mass of 10 g of RSS is slowly added. For this purpose, a little polyvinyl alcohol (PVA) solution is added to the mixture, which serves as a binder to prevent the agglomeration of nanoparticles. Gentle heating of the coated sand results in solvent evaporation, and once the solvent is predominantly evaporated, the coated sand is placed into an oven and dried at 100 °C for 2.5 h. The coated sand is sintered at 500–600 °C for 3 h to improve the adhesion of the Al_2_O_3_ nanoparticles to the silica surface. This methodology ensures a robust, uniform Al_2_O_3_ coating of nanoparticles on the silica sand, making it appropriate for utilization in the adsorption field.

### 2.3 Characterization analysis for SSC–Al_2_O_3_

Various methods, including the analyses of structure and morphology, were employed to investigate physicochemical properties of the prepared SSC–Al_2_O_3_ adsorbent. The specific surface area of the adsorbent was determined using the Brunauer–Emmett–Teller (BET) technique. Elemental composition and chemical components of the material were analyzed using X-ray Fluorescence (XRF; Spectro IQ11/Ametek, Germany). In addition, the elemental mapping on the surface of adsorbents is determined by Energy-Dispersive X-ray Spectroscopy (EDS) and X-ray Diffraction (XRD) for crystallographic structure of SSC–Al_2_O_3_ adsorbent. Scanning Electron Microscope (SEM) was used to investigate the surface morphology and particle structure before and after MTB adsorption. Fourier-Transform Infrared (FTIR) spectroscopy (IR Affinity IS, Shimadzu, Japan) was performed to locate compare the functional groups on the SSC–Al_2_O_3_ surface which are responsible for binding and adsorbing MTB molecules from the aqueous solution.

### 2.4 Optimization of pH and contact time in batch adsorption studies

Batch equilibrium studies assessed the adsorption of MTB onto SSC–Al_2_O_3_, focusing on the effects of pH (2–8) and contact time. The 100 mL of solution with an initial MTB concentration of 50 mg L^−1^ was prepared in 250 mL flasks, each containing 0.5 g of adsorbent. The flasks were shaken at 20 °C and 200 rpm for 75 minutes to reach equilibrium.^37^

### 2.5 Isothermal equilibrium experimentations

The experiment was performed using different masses (0.05, 0.1, 0.2, 0.3, 0.4, 0.5, 0.6, 0.7, 0.8, 0.9, 1.0 g) of SSC–Al_2_O_3_ in 100 mL of MTB aqueous solutions with a concentration of 50 mg L^−1^ at 20 °C, and the pH of MTB solution was adjusted to 6.0 with 0.1 M of HCl or NaOH solution. Equilibrium conditions for the mixture were established by shaking at 200 rpm for 75 min. Afterward, the mixture was filtered through Whatman filter paper no. 42. The concentration of MTB in the aqueous phase at equilibrium can be measured at 664 nm using a UV spectrometer. The equilibrium relationships of the MTB's adsorbed uptake (*U*_e_) onto SSC–Al_2_O_3_ and the equilibrium concentration of MTB in solution were established. The uptake (mg g^−1^) and removal efficiency were calculated based on the equations below:^38,39^4
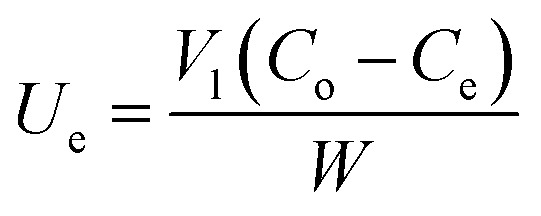
5
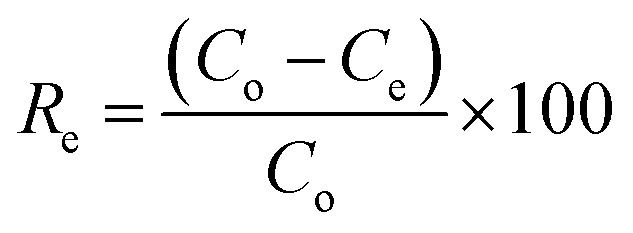
where *C*_o_ and *C*_e_ denote the initial and equilibrium MTB concentrations in the experimental solution, respectively, *V*_1_ represents the solution volume in the flasks, and *W* is the dosage of SSC–Al_2_O_3_. Isothermal adsorption curves were generated by plotting the MTB adsorbed per unit weight of SSC–Al_2_O_3_ (*U*_e_) against the final equilibrium concentration of the contaminant in the solution (*C*_e_). The experimental data obtained from isothermal adsorption studies were analyzed using two widely recognized non-linear adsorption models, Langmuir eqn (6) and Freundlich eqn (7). The STATISTICA Software was used to determine the essential parameters of each model.^40^6
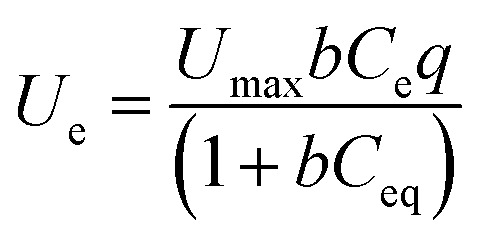

7 *U*_e_ = *K*^T^*C*_eq_^1/*n*^

where *C*_o_ and *C*_eq_ are the initial and equilibrium MTB concentrations in (mg L^−1^). *V*_l_ is the solution volume in (L). *W* is the dosage of the adsorbent in (g). (*U*_e_ and *U*_max_) are the equilibrium uptake (mg g^−1^) of adsorbed MTB onto the adsorbent and the Langmuir maximum adsorption capacity, respectively. *K*_T_ is the Freundlich constant in (L mg^−1^) and *n* is the Freundlich exponent or intensity parameter.

### 2.6 External mass transfer coefficient in batch experiments (*k*_f_)

External mass transfer describes the resistance that MTB molecules face in moving from the bulk to the external surface of the SSC–Al_2_O_3_ particles. This resistance is one of the most crucial aspects affecting the adsorption process since it controls the MTB removal rate from the solution. It is also essential to know and minimize external mass transfer resistance to get optimum adsorption conditions and give some insight into the efficiency of any adsorption process. The external mass transfer coefficient (*k*_f_) can be calculated according to the given relation:8
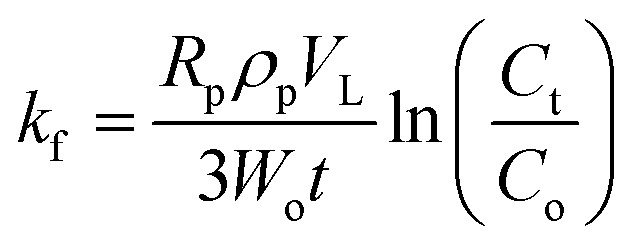
where *R*_p_ and *ρ*_p_ are the particle size and density of SSC–Al_2_O_3_, respectively. *V*_L_ is the volume of the aqueous solution, *W*_o_ is the mass of the used adsorbent, *t* is the contact time, and *C*_t_ and *C*_o_ are the equilibrium and initial concentrations of MTB.^41^

## 3 Continuous column reactor

Dynamic adsorption experiments were performed in a column reactor filled with the SSC–Al_2_O_3_ sorbent material bed. Such a configuration enables the sorbent to be directly interfaced with the new inflow of the MTB dye, allowing for the continuous establishment of a dynamic equilibrium. The column reactor is designed for long operating times under a minimal energy input without significant loss in adsorption performance.^42^ Fixed-bed columns remain the most widely used configuration in industrial adsorption due to their simplicity, low cost, and predictable breakthrough behavior. In contrast, fluidized-bed systems provide enhanced mixing, reduced channeling, and improved mass-transfer rates, making them attractive for high-throughput applications. Several studies have compared these systems for granular adsorbents; however, limited work has evaluated their performance using nano-modified materials. By comparing both systems under identical operating conditions, this study provides practical insight into the influence of hydrodynamics on dye removal efficiency.^43–46^

**Fig. 2:**
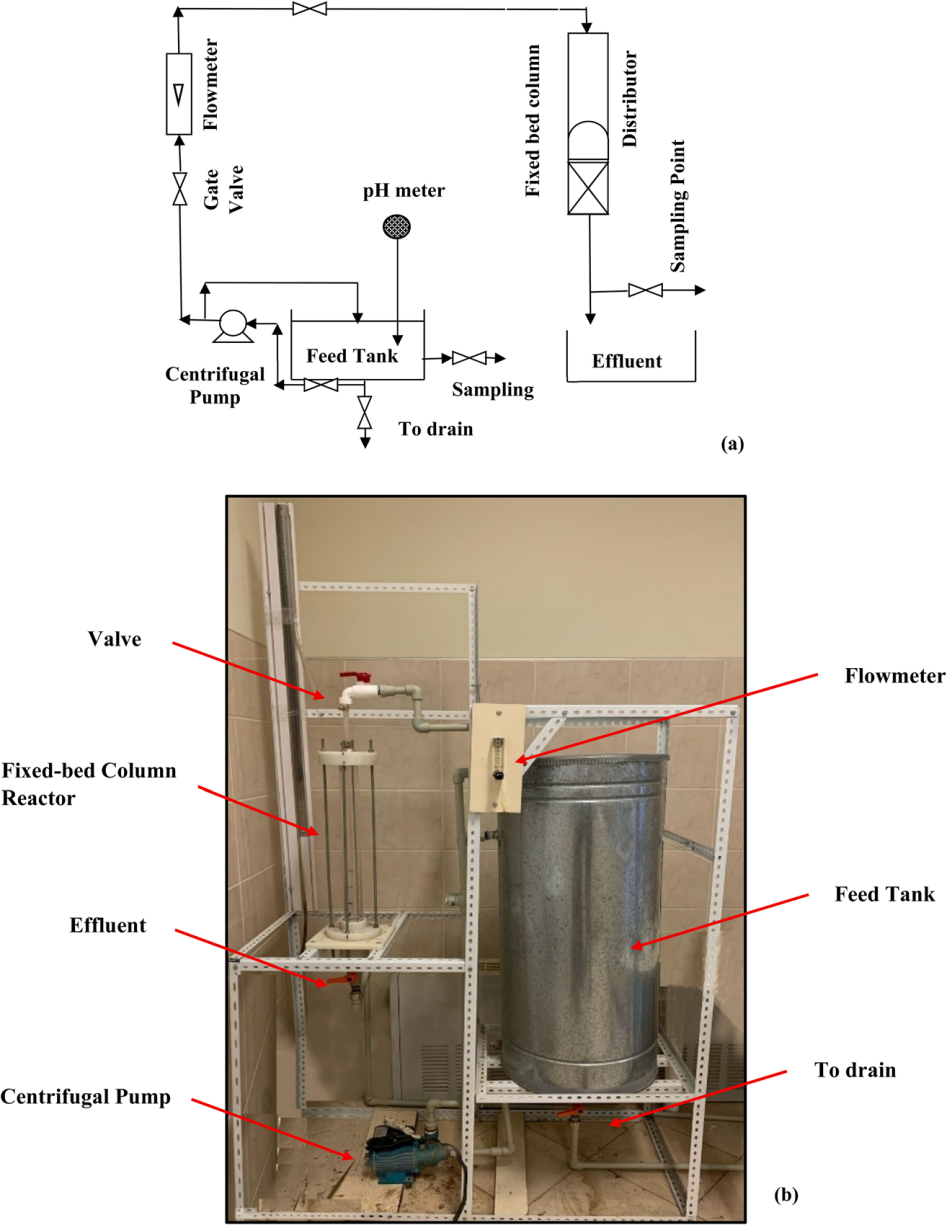
Schematic diagram (a) and pilot-scale setup (b) of the fixed bed column used for the continuous adsorption experiments.

Studies on the adsorption performance of SSC–Al_2_O_3_ in a fixed-bed column for removing MTB molecules from an aqueous solution were carried out. A fixed-bed column reactor was tested to assess its removal capacity and predict the nature of the breakthrough curve during adsorption to obtain an effluent with the lowest MTB dye concentration possible for an extended contact time. A 50 mm × 500 mm (*D* × *H*) adsorption column was packed with SSC–Al_2_O_3_ for the column test. At the top, a distributor was installed to distribute the passage of water in the adsorbent bed evenly. At the bottom of the column, a small mesh sieve was also offered to let the solution (50 mg L^−1^) pass through. The solution's pH in the MTB bioreactor's feed tank was adjusted to the optimal value (6). The column was flushed with deionized water before the adsorption, and the solution was added from the top. A schematic representation of the fixed-bed column configuration is shown in Fig. 2a and the real photo in Fig. 2b. The experiments were carried out for three-bed heights (4 cm = 78.5 g, 7 cm = 137.4 g, and 10 cm = 196.3 g) of SSC–Al_2_O_3_ particles with diameters of (0.6 to 0.9 mm) and a range of flow rates (5, 10, and 15 L h^−1^) of the MTB solution. Adsorption experiments were conducted for continuous periods of 115 to 200 minutes. The concentration of MTB in effluent was measured at regular intervals using a UV spectrophotometer.

The breakthrough data were modelled using a multilayer perceptron (MPL) neural network in IBM SPSS. The input variables were time (*t*), bed height (*L*_s_), and flow rate (*Q*), while the output variable was the normalized concentration (*C*/*C*_0_). The network consisted of one hidden layer with N neurons (optimized between 5 and 15), using the hyperbolic tangent activation function in the hidden layer and a linear activation function in the output layer. Training was performed using the back-propagation algorithm with a 70/15/15 split for training, testing, and validation. The artificial neural network (ANN) represents the nonlinear mapping:9
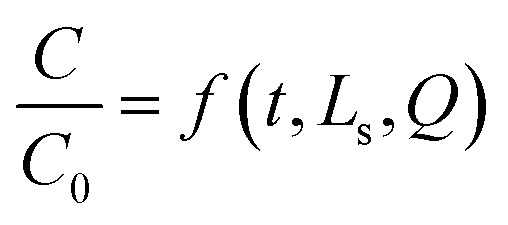
where *f* is defined by the trained network parameters (weights and biases).

## 4 Batch experiment results

Several significant physical properties of SSC–Al_2_O_3_ adsorbent were analyzed in a laboratory to evaluate its direct influence on the effectiveness of MTB adsorption from the solution (Table 1). As depicted in the table, surface area (13.2 m^2^ g^−1^) is crucial for determining the number of available vacant adsorption sites, which also optimizes the adsorption capacity. Furthermore, the solubility of SSC–Al_2_O_3_, which promises stability in aqueous solution, keeps it from dissolving into ions throughout the adsorption process.

**Table 1:** The main physical properties of SSC–Al_2_O_3_ adsorbent

Properties	SSC–Al_2_O_3_ adsorbent
Average particle size (mm)	0.6–0.9
Surface area (m^2^ g^−1^)	13.2036
Pore volume (cm^3^ g^−1^)	0.03026
Porosity	0.44
Color	White
Solubility in water	Insoluble
Morphology	Irregular
Bulk density (g cm^−3^)	1.578
Actual density (g cm^−3^)	2.650

The results of the (XRF) analysis demonstrate that the elemental composition in SSC–Al_2_O_3_ adsorbent contains silica (Si) 53.42%, oxygen (O) 40.37%, and aluminum (Al) 2.85%, as shown in the Table 2. The stoichiometric ratio agrees with the required concentration of aluminum in Al_2_O_3_. This confirms the successful coating of the sand particles with Al_2_O_3_ nanoparticles. Such a large surface area and high chemical purity of the adsorbent are crucial in increasing the adsorption capacity of the adsorbent, especially in the case of MTB wastewater removal.

**Table 2:** Chemical composition of the SSC–Al_2_O_3_ adsorbent

Compounds	Weight %	Compounds	Weight %
Si	53.42	Ti (trace)	0.22
O	40.37	Mn (trace)	0.13
Al	2.85	Mg (trace)	0.62
Ca (trace)	0.85	K (trace)	0.42
Fe (trace)	1.10	P (trace)	0.15
Na (trace)	0.28	Cl (trace)	0.12

**Fig. 3:**
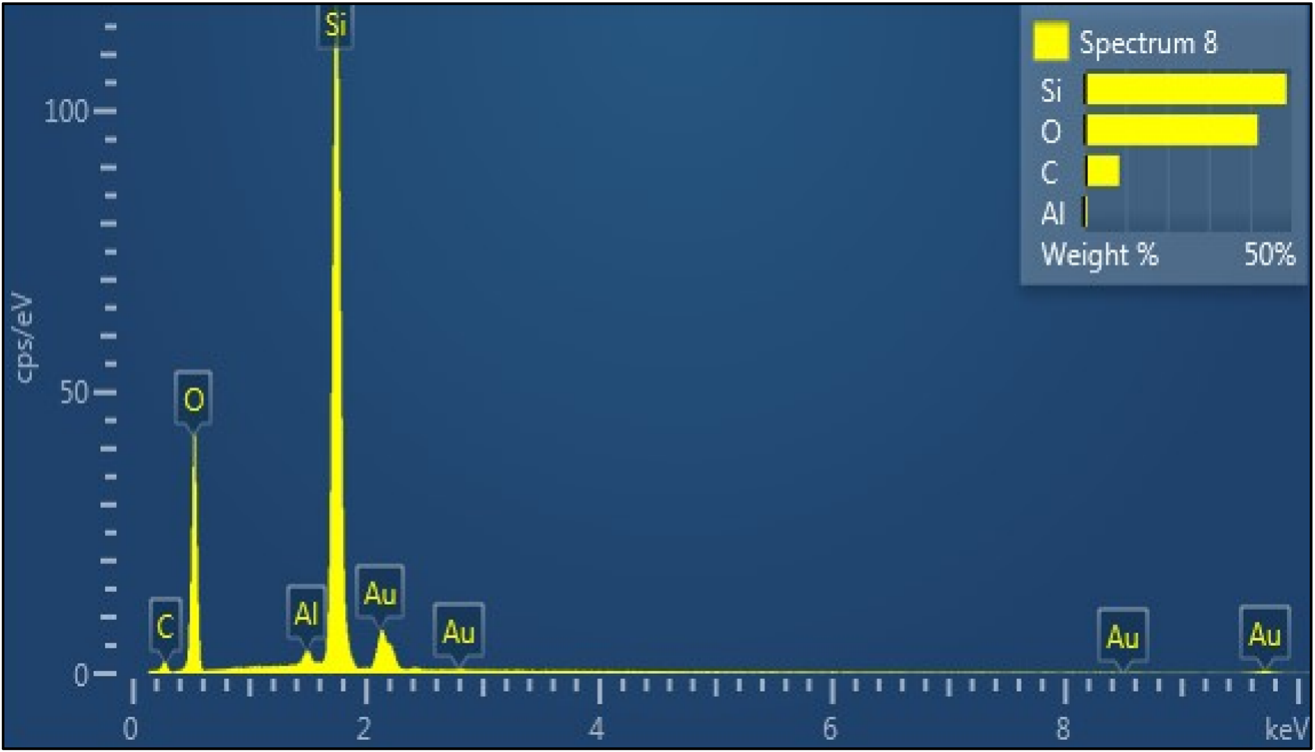
EDS Spectrum and elemental composition of SSC–Al_2_O_3_ adsorbent.

Results of the EDS spectrum scan, portrayed in Fig. 3, exhibit the elements present in the adsorbent composition, namely silicon (Si) and oxygen (O), in addition to (Al), carbon (C), and gold (Au). This indicates successful coating to maximize adsorption and the relative presence of silicon and aluminum as the constituents of the SSC–Al_2_O_3_ nanoparticles. Such structures increase surface area, provide active sites for MTB adsorption, and enhance the total sorption capacity.^47^ Small peaks for carbon and gold probably indicate contamination and do not substantially affect performance. The elemental ratio reflects the adsorbent efficiency in terms of adsorption.

**Fig. 4:**
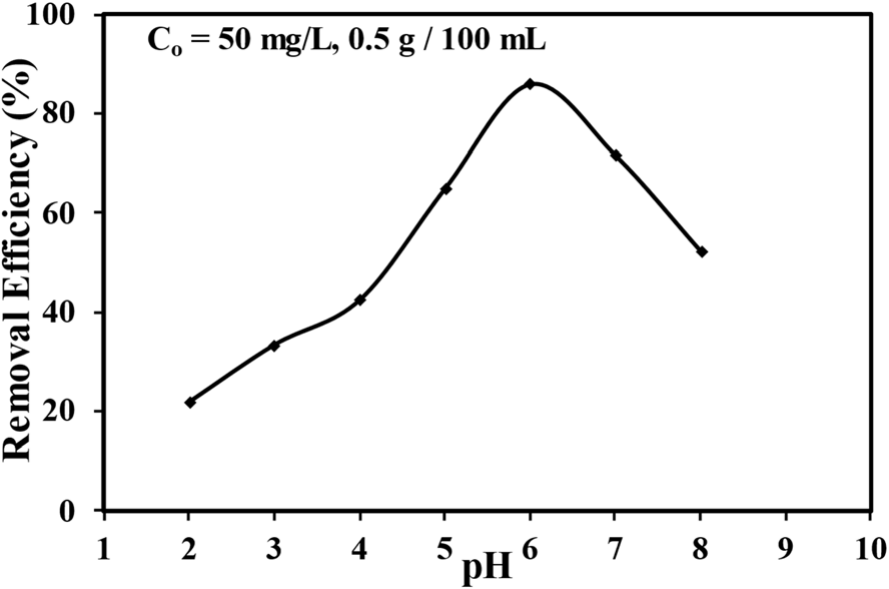
Impact of pH on the adsorption removal percentage of MTB dye in aqueous solution.

The effect of initial pH on the removal of MTB dye is presented in Fig. 4. The pH of the solution can significantly influence the adsorption of MTB dye because the surface charge of the adsorbent and the chemical characteristics of the dye are associated with pH.^48,49^ The low adsorption efficiency at low pH is due to the protonation of the surface of the adsorbent, which leads to a positive charge that repulses the cationic MTB dye molecules, thereby limiting their adsorption capacity.^50^ The maximum adsorption was observed at pH 6, indicating favorable conditions for adsorption. Due to that, the surface charge of SSC–Al_2_O_3_ is more neutral, leading to maximum dye molecule interaction and adsorption.^51^ However, above pH 6, the removal efficiency starts sagging. The reduction in adsorption at higher pH is likely due to the adsorbent surface acquiring a negative charge, resulting in an electrostatic repulsion between the negatively charged adsorbent and dye molecules, in conjunction with increased competition for adsorption sites by hydroxyl ions (OH^−^).^52,53^

**Fig. 5:**
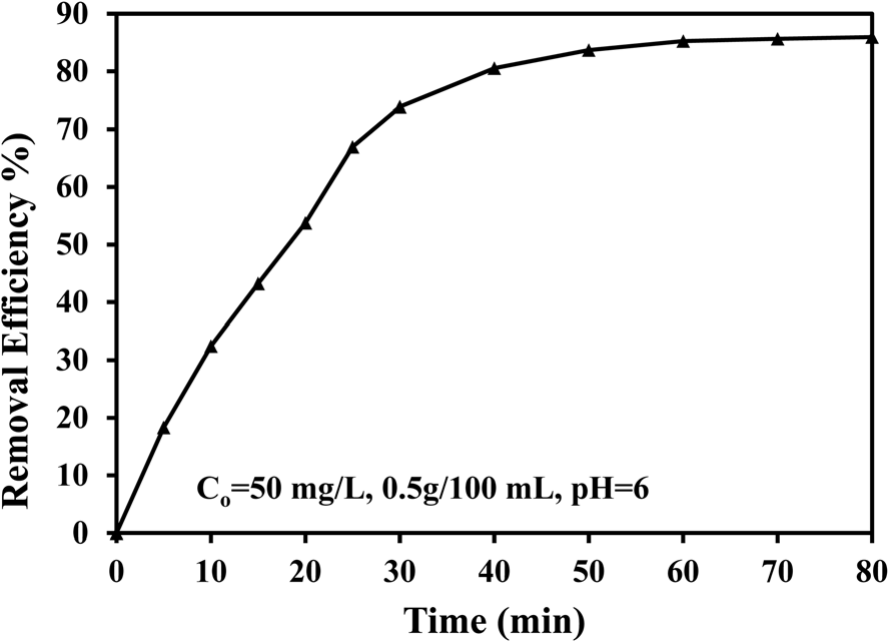
Impact of extended contact time for MTB adsorption efficiency using SSC–Al_2_O_3_ adsorbent.

The adsorption removal of MTB dye at different contact times is shown in Fig. 5. Since SSC–Al_2_O_3_ has a large number of vacancy adsorption sites on the surface, adsorption is rapid in the initial stage of the adsorption process (as shown in the Fig. 5). When introducing the dye into the solution, a steep concentration gradient is established due to many active sites of the SSC–Al_2_O_3_ surface possibly being occupied by the dye molecules. This gradient ensures a fast and effective interface adsorption process, wherein the dye molecules quickly settle at the rich sites. During this initial stage, the driving force for adsorption is especially significant because of the high concentration of vacant sites.^54^ The adsorption process led to a gradual more dye molecules being adsorbed on the adsorbent, filling available sites. This decreases the number of available adsorption sites and the concentration gradient between the solution and the adsorbent surface, resulting in a decrease in the adsorption rate. This step is known as the saturation stage, at which point the adsorbent surface is covered with dye molecules, leading to a steep decrease in the adsorption rate when the available sites are limited.^55^ Thus, a contact time of 75 min was found suitable for obtaining equilibrium adsorption in the following experiments.

**Fig. 6:**
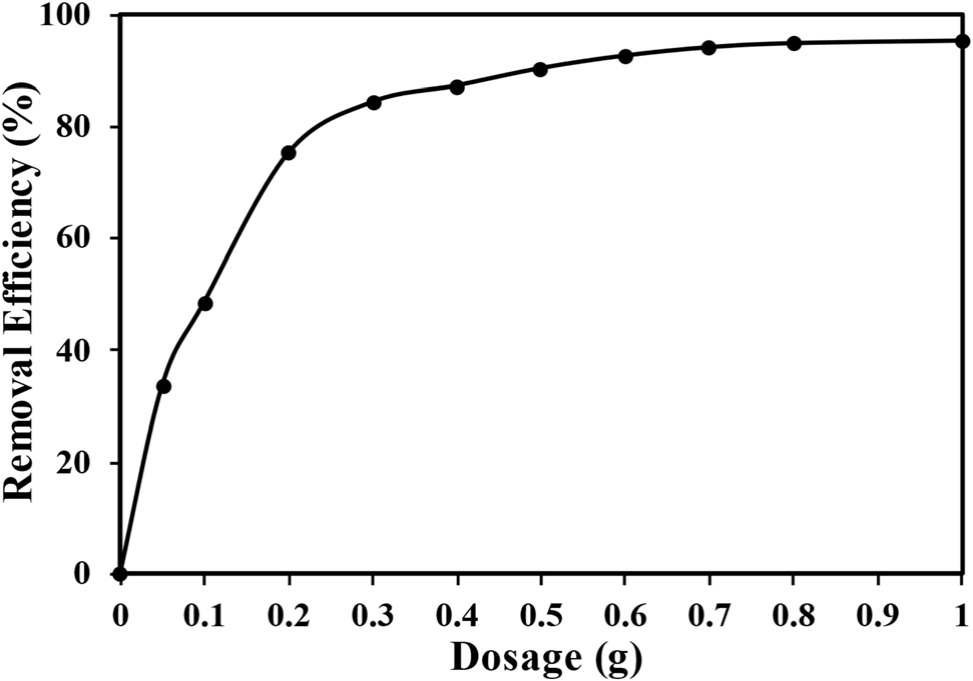
The impact of various dosages of SSC–Al_2_O_3_ on the interaction of MTB molecules.

Fig. 6 demonstrates that the adsorption removal of MTB dye from a solution increase as the dosage of SSC–Al_2_O_3_ adsorbent is enhanced. This phenomenon is due to the increase in available surface area for adsorption. This provides extra binding sites for MTB dye molecules, which promotes adsorption effectiveness. Furthermore, a higher dosage causes an increase in the number of readily available adsorption sites on the adsorbent surface. This indicates that the adsorbent can hold more MTB dye molecules, resulting in better removal efficiency. Moreover, raising the dose facilitates the mass transfer of dye molecules from the solution to the adsorbent.^56,57^ The maximum removal efficiency of MTB onto SSC–Al_2_O_3_ reached 85.90% within 75 minutes of contact time.

In the adsorption mechanism, the interaction between Al_2_O_3_ nanoparticles and MTB molecules can be explained by electrostatic attraction and surface complexation. The Al_2_O_3_ surface provides negatively charged and energetically active hydroxyl groups, which facilitate binding with the cationic MTB dye through ion-dipole and hydrogen bonding interactions. Furthermore, the porous structure of the silica sand substrate enhances diffusion and provides multiple vacant sites for adsorption, particularly during the initial stages. These molecular-level interactions account for the observed high adsorption capacity and sharp breakthrough profiles in the fixed-bed column, linking the chemical properties of Al_2_O_3_–MTB binding directly to the experimental results.

**Fig. 7:**
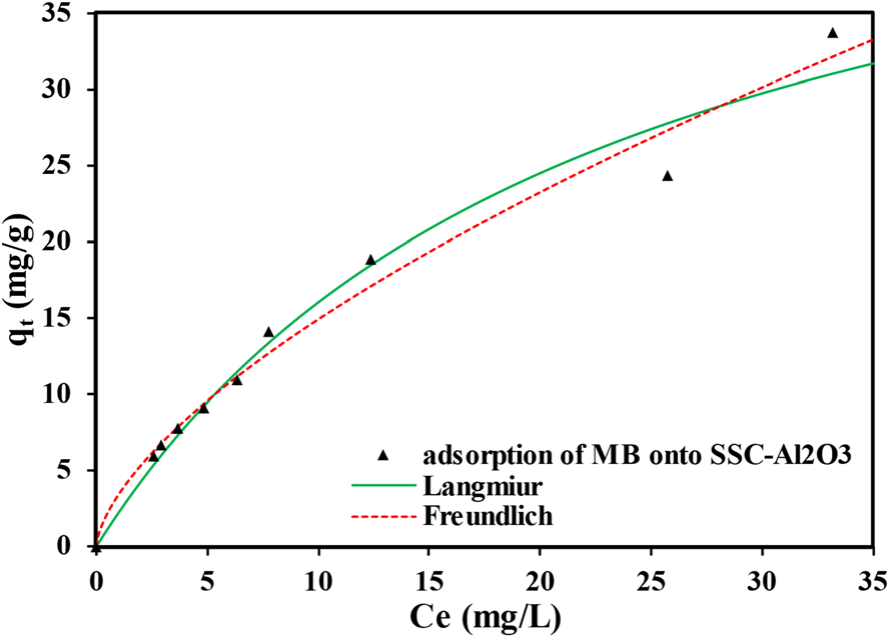
Equilibrium sorption isotherm plot of MTB adsorption onto SSC–Al_2_O_3_ adsorbent.

The results also confirmed that the SSC–Al_2_O_3_ adsorbent exhibited a high level of consistency with the Freundlich isotherm model for the adsorption of MTB molecules, with a remarkable determination coefficient of 0.9817, as shown in Fig. 7 and Table 3. This suggests that the adsorption occurs on a surface with varying energies and indicates favorable interactions between the MTB molecules and the adsorbent, reflecting multilayer adsorption. The value of (*K* = 3.415) suggests a higher affinity between the MTB and the adsorbent, indicating stronger adsorption. Freundlich exponent (*n* = 1.562) provides information on surface heterogeneity.^58^ Detailed model parameters derived from STATISTICA Software applications are presented in Table 3. The maximum adsorption capabilities of several adsorbents for MTB dye removal are shown in Table 4.

**Table 3:** Isotherm adsorption parameters for MTB molecule binding to the SSC–Al_2_O_3_ adsorbent

Model	Parameter	MTB
	*U*_m_ (mg g^−1^)	52.097
Langmuir	*b* (L mg^−1^)	0.0446
	*R* ^2^	0.9785
	*K*_T_	3.415
Freundlich	*n*	1.5620
	*R* ^2^	0.9817

**Table 4:** Adsorption capacities of various adsorbents for MTB removal from aqueous solutions

Adsorbent	*q*_max_ (mg g^−1^)	Ref.
SSC–Al_2_O_3_	34.25	Current study
Clay	300	59
Diatomite clay	198	59
Bentonite clay	150	59
Montmorillonite clay	289.12	59
Natural clay	27.78	7
Spent activated clay	127.5	59
Fly ash geopolymer	18.3	31
Monolithic algal green powder	126.79	60
Glass wool	2.24	4 and 59
Rice husk	40.6	61 and 62
Esterifying wheat straw	312.5	63 and 64
Geopolymer silicate bricks	77.34	7
Fe_3_O_4_ metakaolin geopolymer	76.34	31
Phoenix tree leaf	80.9	61 and 63
Wood apple shell	95.2	65
Cereal chaff	20.3	61 and 66
Iron oxide magnetic biochar	46.2	67 and 68
Commercially activated carbons	163.7	69
Kaolin geopolymer	4.75	31
Fly ash	4.47	59
Wood apple shell	95.2	63 and 65
Hair	120	59
Foamed metakaolin geopolymer	39.5	31
*Casuarina equisetifolia* pines	41.35	70
Spent tea leaves	300.052	71
Living biomass	1.17	59
Chitosan-corncob lignin biochar	499.8	67
Lotus leaf	241.4	63
Crushed brick	96.61	59 and 72
Water hyacinth root	185	61 and 73

The results also confirmed that the value of *k*_f_ determined based on eqn (8) was (2.086 × 10^−5^ m s^−1^). This denotes a moderately high mass transfer rate, implying efficient movement of MTB molecules from the bulk solution to the adsorbent surface. Moreover, this value highlights the critical need to optimize the adsorbent's surface characteristics and experimental settings to boost adsorption efficiency.^74^

**Fig. 8:**
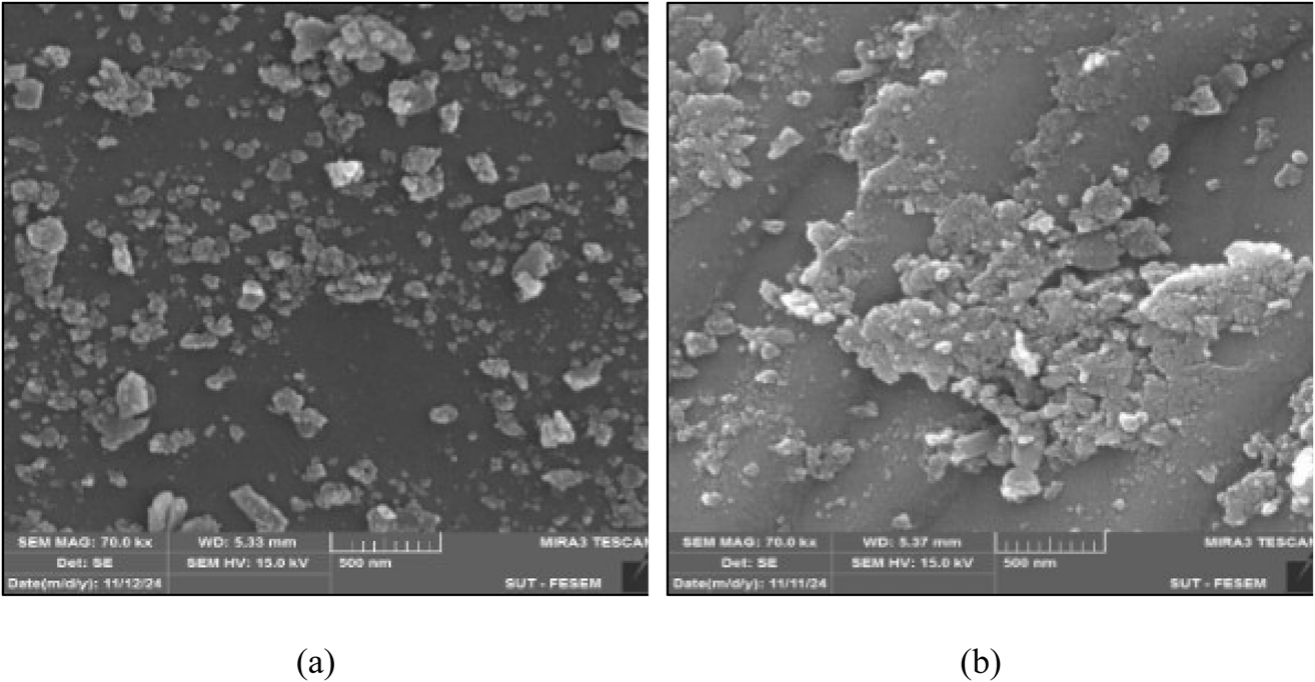
Scanning electronic microscope for (a) unloaded MTB, (b) loaded MTB onto SSC–Al_2_O_3_.

Fig. 8a and b depict SEM microscopic images of the SSC–Al_2_O_3_ adsorbent before and after MTB adsorption. The SEM scan image shows the surface morphology in various sizes and shapes. The scan images reveal significant changes in surface morphology. Initially, the surface is rough and irregular, indicating a high surface area and numerous binding sites. After adsorption, a smoother surface layer is observed, confirming MTB dye's adherence to the adsorbent. This transition demonstrates the SSC–Al_2_O_3_ adsorbent's effectiveness as an adsorbent, with ample sites for sorption and strong interaction between the dye molecules and the adsorbent surface.

**Fig. 9:**
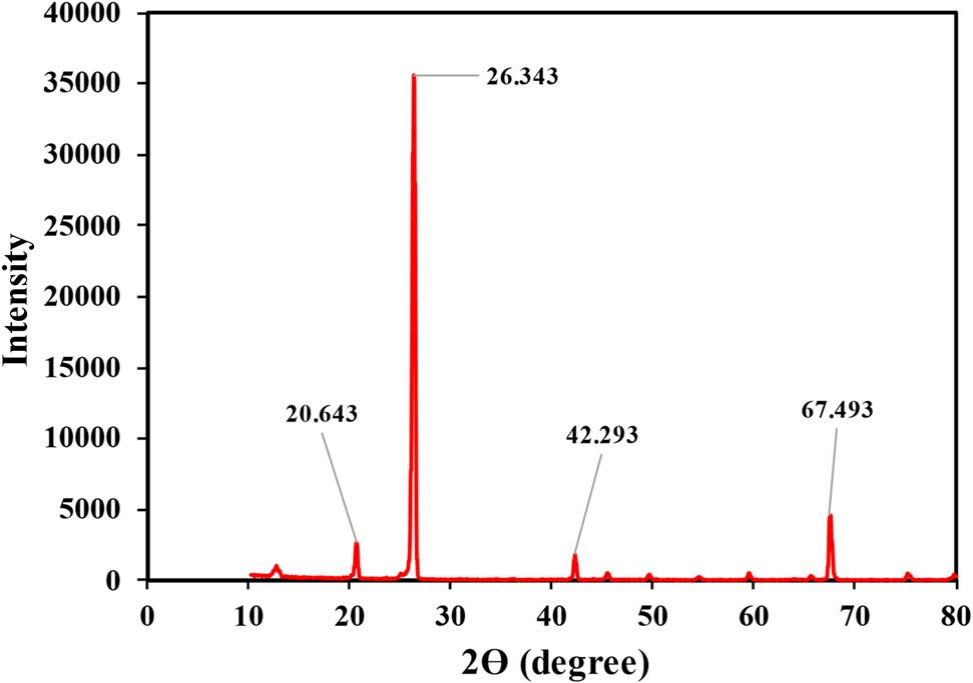
XRD analysis of SSC–Al_2_O_3_ nanoparticles for MTB adsorption.

The XRD patterns of SSC–Al_2_O_3_ nanoparticles in the adsorption of MTB dye are shown in Fig. 9. The figure shows multiple distinct peaks at various values of 2*θ*. The sharp peak corresponding to 26.343° infers the silica sand to be crystalline, and other considerable peaks at 20.643°, 42.293°, and 67.493° confirm the appearance of Al_2_O_3_ nanoparticles on the adsorbent surface. This agrees with the peaks that are of high intensity, with a maximum of 35 532 counts, indicating that Al_2_O_3_ nanoparticles have successfully coated silica sand. The active sites available for anchoring MTB dye molecules are increased due to this coating, which also improves the adsorption properties.^75^

**Fig. 10:**
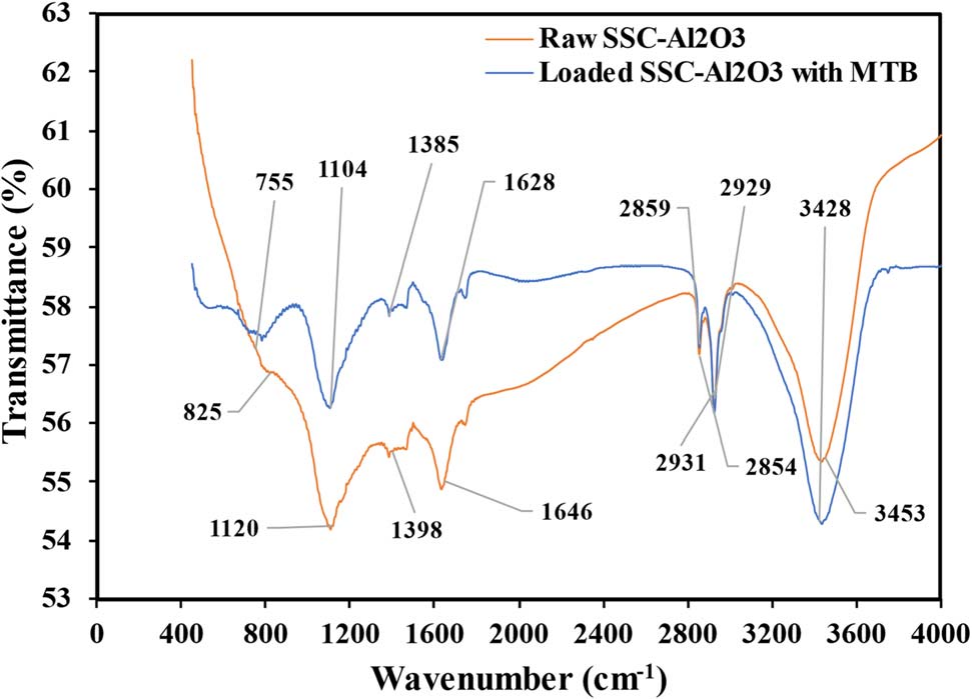
FTIR spectra of SSC–Al_2_O_3_ before and after loaded with MTB molecules.

FTIR spectra of the synthesized SSC–Al_2_O_3_ before and after adsorption of MTB molecules were measured in a wavenumber range of 4000–400 cm^−1^ as shown in Fig. 10. Various functional groups were identified on the surface of SSC–Al_2_O_3_ by the FTIR scan that positively affects SSC–Al_2_O_3_ adsorption properties. The SSC–Al_2_O_3_ adsorbent spectra before and after loading with MTB revealed notable differences in some peaks. These changes indicate the binding of MTB molecules to specific functional groups on the adsorbent surface.

The analysis scan shows multiple prominent peaks, associated with functional groups on the SSC–Al_2_O_3_ surface. The peaks of the spectra before loading with MTB were found at 825 cm^−1^ (Si–O–Si bending vibrations), 1120 cm^−1^ (Si–O stretching vibrations), 1398 cm^−1^ (C–H bending vibrations), 1646 cm^−1^ (bending vibrations of adsorbed water molecules), 2854 cm^−1^ and 2931 cm^−1^ (C–H stretching vibrations), and 3453 cm^−1^ (O–H stretching vibrations).^76^ This suggests that MTB molecules interact with the functional groups of the adsorbent, resulting in significant changes after MTB adsorption. The most substantial changes of interest are the peaks at 1385 cm^−1^, 1628 cm^−1^, 2859 cm^−1^, and 2929 cm^−1^, which indicate the potential entrapment of hydroxyl (–OH), carboxyl (–COOH), and possibly amine (–NH_2_) groups around MTB molecules.^77^ These interactions improve the adsorbent's adsorption properties, highlighting its capability to trap MTB dye molecules and the significance of these functional groups in the adsorption process.

## 5 Continuous process results

**Fig. 11:**
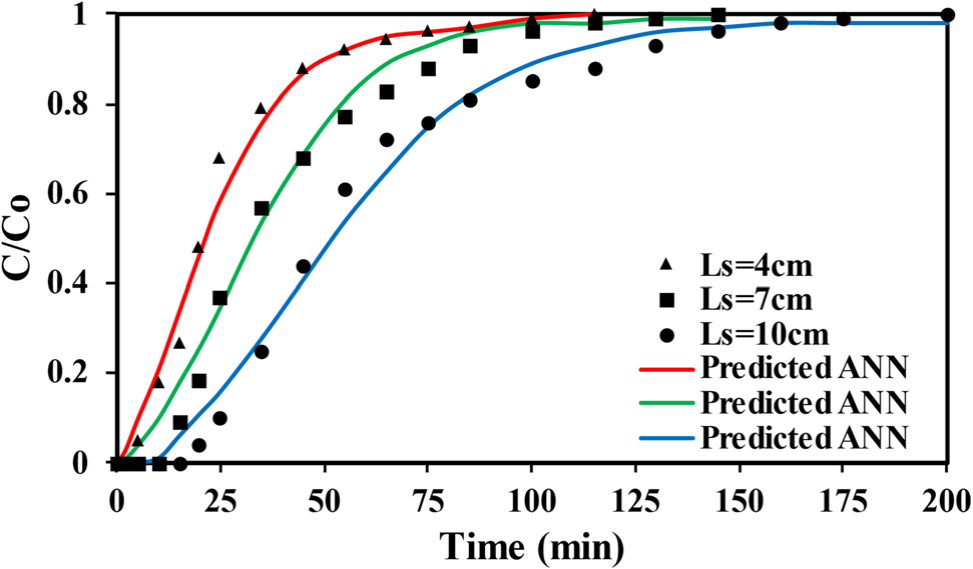
Experimental and theoretical breakthrough curves for MTB adsorption at various bed depths (*C*_o_ = 50 mg L^−1^, *Q* = 10 L h^−1^, d*p* = 0.6–0.9 mm diameter).

The performance of the SSC–Al_2_O_3_ adsorbent in the fixed-bed column was evaluated under different operating conditions. Fig. 11 demonstrates that the breakthrough time for achieving equilibrium with MTB dye decreases when smaller bed heights are used in the fixed bed column. This reduction is attributed to the fewer adsorption sites available, leading to a diminished surface area for interaction between the MTB and the active sites of the SSC–Al_2_O_3_ adsorbent.^78,79^ The figure also indicates that a bed height of 10 cm provides the longest duration to reach the breakpoint (200 minutes), whereas a bed height of 4 cm results in the shortest duration (115 minutes). Consequently, increasing the depth of the SSC–Al_2_O_3_ bed in the column enhances the retention time of the dye solution within the column. This extended contact duration facilitates more effective adsorption, as the dye molecules have a prolonged opportunity to interact with the adsorbent. This scenario may also lead to slower mass transfer of dye molecules from the bulk solution to the surface of the adsorbent.^80,81^

**Fig. 12:**
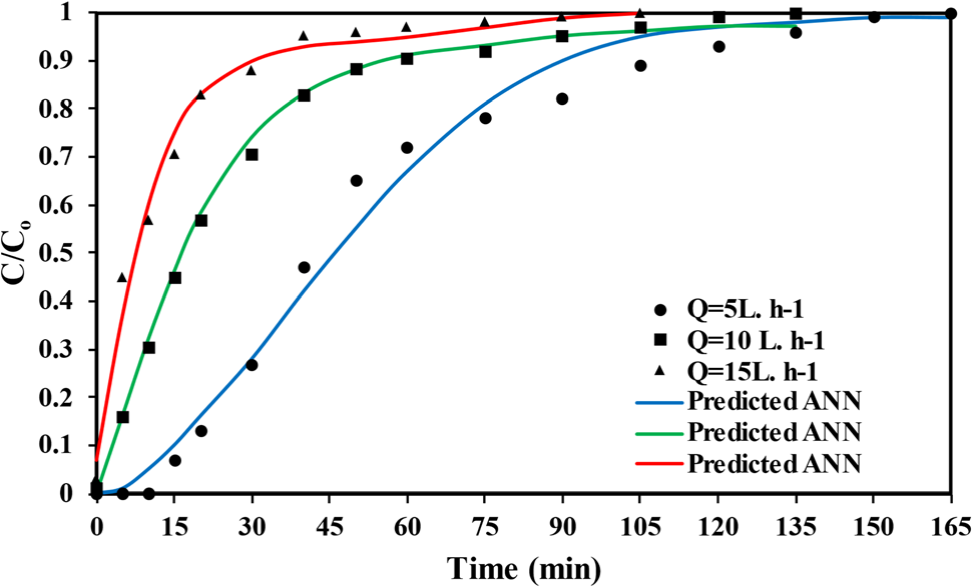
Experimental data and theoretical breakthrough curves for adsorption of MTB at various flowrates (*C*_o_ = 50 mg L^−1^, 7 cm bed height, d*p* = 0.6–0.9 mm diameter).

Increasing the flow rate of the MTB dye solution from 5 L h^−1^ to 10 L h^−1^ or 15 L h^−1^ through the bed particles, as shown in Fig. 12, results in insufficient contact time between the MTB dye and the active sites of the SSC–Al_2_O_3_ adsorbent. This leads to a reduced uptake of MTB dye, a noticeable decrease in adsorption capacity, steeper breakthrough curves, and a significant reduction in the breakpoint due to high intra-particle resistance. These conditions may hinder effective adsorption and potentially lower the overall removal efficiency.^82,83^ The breakpoint time decreases from 165 minutes at 5 L h^−1^ to 135 minutes at 10 L h^−1^ and 105 minutes at 15 L h^−1^. Furthermore, increasing the flow rate causes uneven dispersion of the MTB dye solution within the bed column, potentially leading to a faster saturation of certain areas with dye molecules and incomplete utilization of the column's overall adsorption capacity.^84^ However, increasing the dye solution's flow rate through the column can reduce the thickness of the boundary layer film around the SSC–Al_2_O_3_ particles, thus decreasing the resistance to mass transfer of MTB molecules to the adsorbent surface.^85^Fig. 11 and 12 also show that the experimental breakthrough data for MTB molecule adsorption closely matched the predicted data obtained using the ANN in IBM SPSS. Table 5 presents a comparison of the maximum column adsorption capacities of several adsorbents for MTB removal, emphasizing the efficacy of the SSC–Al_2_O_3_ used in the current study.

**Table 5:** Comparison of maximum column adsorption capacities of various adsorbents for different pollutants

Adsorbents	Pollutants	*q*_max_ (mg g^−1^)	References
SSC–Al_2_O_3_	MTB	34.25	Current study
Quartz sand	MTB	11.96	33
Ultrasonic surface modified chitosan supported on sand	MTB	51.8	86
Sand	MTB	5.31	33 and 87
Graphite oxide coated sand	MTB	55.33	87
Granular NaX zeolite/attapulgite	MTB	11.51	1
Sand	MTB	0.138	88
Ceramsite media	MTB	39.56	33
Foamed geopolymer	MTB	7.30	31
nZVI-coated biochar	MTB	118.056	6
Biochar and Kaolin	MTB	20.06	31 and 89
Waste watermelon rind	MTB	113.5	6 and 81
Modified rice husk	MTB	101.3	31 and 90
Pine cone	MTB	55.68	31 and 32
Fly ash geopolymer	MTB	7.80	31 and 91
NaOH-modified rice husk	Malachite green	101.31	6 and 81
Tamarind seed powder	Acid yellow 17	978.5	81
Sand	Lead(ii)	4.0	87
Graphite oxide coated sand	Lead(ii)	46.83	87
KOH-activated carbonized argan shell	Amoxicillin	87.48	92
*E coli* biofilm supported on KOH-activated carbonized argan shell	Amoxicillin	122.89	92
KOH-activated carbonized argan shell	Paracetamol	110.73	92
*E coli* biofilm supported on KOH-activated carbonized argan shell	Paracetamol	128.81	92
Modified guazuma ulmifolia biochar	Ketoprofen	307.81	93
MgO–Al_2_O_3_ modified rice husk biochar	Trimethoprim	14	94
	Acetaminophen	5.0	
Nano ZrO_2_/Al-sludge composite	Fluoride	1.01	95

The adsorption results were quantitatively analyzed through breakthrough curves received from the fixed-bed column process, adsorption capacities, and efficiency calculations. The mechanistic interpretation was further supported by FTIR evidence, which revealed shifts in Al–O and O–H bands after MTB adsorption, confirming the involvement of specific functional groups. Moreover, the relatively low surface area (13.2 m^2^ g^−1^) reflects the composite design, where Al_2_O_3_ nanoparticles are immobilized on coarse silica sand rather than existing as free nano powders. While this reduces BET values compared to pure nanostructured alumina, it provides mechanical stability and scalability for fixed-bed use. Notably, despite the lower surface are, the adsorbent showed high uptake capacity and sharp breakthrough curves, confirming that adsorption efficiency is governed by the accessibility of active functional groups rather than surface area alone.

The ANN model demonstrated strong predictive capability for the fixed-bed adsorption system. For the breakthrough curves obtained at different bed depths, the ANN achieved an *R*^2^ = 0.9656, indicating excellent agreement between the experimental data and the theoretical predictions. Similarly, for the breakthrough curves generated at different flow rates, the ANN model achieved an *R*^2^ = 0.9656, confirming its ability to accurately capture the nonlinear dynamics of MTB transport through the packed column. These high correlation values show that the ANN successfully learned the complex relationship between operational parameters (bed height, flow rate, and time) and the resulting breakthrough behavior, producing predictions that closely follow the experimental trends.

## 6 Conclusions

The SSC–Al_2_O_3_ adsorbent was found to be highly effective for MTB dye removal in both batch and fixed bed reactors, with low resistance to mass transfer due to its strong adsorption capacity. This efficiency is attributed to the presence of negative and energetic functional groups that bind MTB molecules and the availability of vacant sites, particularly during the initial adsorption stage. Freundlich's model efficiently depicted the sorption behavior of MTB molecules onto the adsorbent. Breakthrough curves in fixed-bed columns showed sharper profiles at higher flow rates and lower bed heights, while the mass transfer zone expanded with increasing bed height, confirming predictable adsorption dynamics. These findings demonstrate that SSC–Al_2_O_3_ is economically feasible and reliable for industrial wastewater treatment applications. The integration of batch and fixed-bed evaluations provides practical insights into the behavior of the nanocomposite under realistic operating conditions, offering a sustainable and economically viable alternative to conventional high-cost adsorbents. Therefore, this work contributes to innovative waste-to-resource materials engineering and practical advancements in sustainable wastewater treatment technologies.

## Author contributions

E. B. Hussein: conceptualization, methodology; software; formal analysis; investigation; sampling; samples preparation; validation; writing original draft; writing – review & editing. F. A. Rasheed: supervision; project administration; validation; writing – review & editing.

## Conflicts of interest

The authors declare no conflict of interest in publishing this paper.

## Data Availability

The data that support the findings of this study are available from the corresponding author upon reasonable request.
